# A phase II trial of stereotactic body radiotherapy in 4 fractions for patients with localized prostate cancer

**DOI:** 10.1186/s13014-022-02037-y

**Published:** 2022-04-04

**Authors:** S. Kawakami, H. Tsumura, T. Satoh, K. Tabata, A. Sekiguchi, T. Kainuma, M. Nakano, M. Iwamura, H. Ishiyama

**Affiliations:** 1 Department of Radiation Oncology, Kitasato University School of Medicine, 1-15-1 Kitasato, Minamiku, Sagamihara, Japan; 2 Department of Urology, Kitasato University School of Medicine, 1-15-1 Kitasato, Minamiku, Sagamihara, Japan

## Abstract

**Purpose/objective(s):**

To report results from our phase II study of stereotactic body radiotherapy (SBRT) delivering 36 Gy in 4 fractions for patients with localized prostate cancer.

**Materials/methods:**

We enrolled 55 patients treated with SBRT delivering 36 Gy in 4 fractions between 2015 to 2018. All patients were categorized as low-risk (n = 4), intermediate-risk (n = 31) or high-risk (n = 20) according to National Comprehensive Cancer Network criteria. Median age was 73 years (range 54–86 years). Two-thirds of patients (n = 37) had received androgen-deprivation therapy for 3–46 months (median, 31 months). Median duration of follow-up was 36 months (range 1–54 months). We used Radiation Therapy Oncology Group and National Cancer Institute—Common Toxicity Criteria version 4 for toxicity assessments. Quality of life (QOL) outcomes were also evaluated using the Expanded Prostate Cancer Index Composite (EPIC).

**Results:**

Protocol treatments were completed for all patients. Six patients experienced biochemical failures. Among these six patients, three patients experienced clinical failure. One patient showed bone metastasis before biochemical failure. One patient died of gastric cancer. The 3-year biochemical control rate was 89.8%. Acute grade 2 genitourinary (GU) and gastrointestinal (GI) toxicities were observed in 5 patients (9%) and 6 patients (11%), respectively. No grade 3 or higher acute toxicities were observed. Late grade 2 GU and GI toxicities were observed in 7 patients (13%) and 4 patients (7%), respectively. Late grade 3 GU and GI toxicities were observed in 1 patient (1.8%) each. EPIC scores decreased slightly during the acute phase and recovered within 3 months after treatment.

**Conclusion:**

Our phase II study showed that SBRT delivering 36 Gy in 4 fractions was safe and effective with favorable QOL outcomes, although this regimen showed slightly more severe toxicities compared to current standards.

## Background

According to the forecast for 2020, prostate cancer will be the most common male cancer in Japan. In particular, numbers of morbidities and deaths from prostate cancer among elderly individuals ≥ 75 years old are expected to increase. Stereotactic body radiotherapy (SBRT) is a method that can cure prostate cancer efficiently with limited medical resources (radiotherapy equipment and human resources) and needs are expected to increase further.

Although the majority of reported series have used 35–37 Gy in 5 fractions for SBRT [1–3], the optimal size and number of fractions have not yet been established. For examples, Alongi et al. described their phase II study which irradiated 35 or 37.5 Gy in five consecutive fractions and its feasibility [1]. Katz et al. reported their SBRT experience using Cyberknife irradiating 35 or 36.25 Gy in 5 fractions and median follow-up of 9 years. Meanwhile, Fuller et al. reported their experience with 38 Gy in 4 fractions using brachytherapy-like dosimetry [4, 5]. Kang et al. described their experience with 32–36 Gy in 4 fractions and median follow-up of 4 years [6]. We have previously reported the results from our Phase I dose-escalation study of SBRT using 4 fractions, which recommended 36 Gy in 4 fractions [7].

In this study, we report results from our Phase II study of SBRT using 4 fractions for patients with localized prostate cancer.

## Materials & methods

Eligible patients had to have histologically confirmed adenocarcinoma of the prostate with clinical stage T1–T3b with neither lymph node nor distant metastases according to the Union for International Cancer Control (UICC) TNM classification version 7. Pelvic MRI scans were implemented for all patients before treatment and all major and minor suggestions from the scans were reflected to their risk classification.

Eligibility also required that patients be ≥ 20 years old with Eastern Cooperative Oncology Group performance status 0–1. Exclusion criteria for this study were: (1) history of pelvic radiotherapy; (2) deteriorated organ functions; (3) poorly controlled diabetes mellitus; (4) acute inflammatory disease; (5) psychiatric disorder; or (6) continuing administration of steroidal drugs. Pretreatment evaluations included chest radiography, computed tomography (CT) of the abdomen and pelvis, and magnetic resonance imaging of pelvis.

All patients were treated by image-guided intensity-modulated radiotherapy using tomotherapy. The four-fractionated treatments were scheduled from Thursday to Tuesday with a two-day break of Saturday and Sunday. All patients were implanted with fiducial markers at the apex and base of the prostate before CT simulation. They were also asked to empty rectum and bladder, then 80 cc of saline was installed into their bladder just before CT simulation and radiotherapy sessions. Clinical target volume (CTV) covered the prostate gland and proximal 1 cm of the seminal vesicles. Planning target volume (PTV) was defined as the CTV plus 5-mm margins except posteriorly (3-mm). Prescribed dose was delivered to at least 95% of the PTV. Outer circumference of the rectum was delineated from the rectosigmoid junction to the caudal edge of the ischium or 3 cm above the anal verge, whichever was lower. Outer circumferences of the bladder, femoral head, and small intestine (if this was close to the PTV) were also delineated. Dose-volume constraints for normal tissues were calculated from guidelines for conventional fractionation experiences [8, 9] (Table 1). Hydrogel spacer was not utilized in this study.

**Table 1:** Dose-volume constraints for normal tissues

Normal tissue dose-volume	Constraint
Rectum	
V31 Gy	25%
V28 Gy	40%
V24 Gy	55%
V20 Gy	65%
Bladder	
V28 Gy	30%
V24 Gy	50%
Femoral* head*	
Maximum	28 Gy
Small intestine	
Maximum	24 Gy

V, volume

Generally, in this study, low-risk patients according to National Comprehensive Cancer Network criteria (NCCN) were treated with radiotherapy alone. Intermediate-risk patients underwent 6 months of neoadjuvant androgen deprivation therapy (ADT) before radiotherapy. High-risk patients initially underwent 6 months of neoadjuvant ADT, and adjuvant ADT was continued for 36 months after completion of radiotherapy.

Adverse events were evaluated according to the National Cancer Institute’s Common Terminology Criteria for Adverse Events version 4.0 and the Radiation Therapy Oncology Group scale [10]. In addition, the Expanded Prostate Cancer Index Composite (EPIC) [11] was used for assessment of health-related quality of life. Follow-up evaluations were performed at 1, 3, 6, 9, and 12 months until 1 year after treatment, and at 6-month intervals thereafter.

Overall survival was calculated using the Kaplan–Meier method. Biochemical failure was defined according to the Phoenix ASTRO consensus (Nadir + 2) [12].

The primary endpoint of the study was biochemical disease-free survival rate of 4-fractionated 36 Gy SBRT, and secondary endpoint was treatment related toxicity. We calculated the sample size expecting a ≥ Grade 2 toxicity rate of 15%, with a threshold of 30%. With the alpha and beta error levels set at 0.05 and 0.2, respectively, the required number of eligible patients was 50. We finally decided on a sample size of 55, including ineligible patients.

Statistical analyses were performed using R version 3.5.1 software (R Project for Statistical Computing, Vienna, Austria).

## Results

Fifty-five patients were recruited and finished planned treatments. Patient characteristics are shown in Table 2. Median follow-up was 36 months (range 1–54 months). Two-thirds of patients received hormonal therapy for 3–46 months (median, 31 months). Protocol treatment was completed for all patients.

**Table 2:** Patient characteristics

Variables	Values	SD
Age (years)	72	6.84
iPSA (ng/mL)	14.64	15.66
Gleason score		
3 + 3	9	
3 + 4	21	
4 + 3	8	
4 + 4	10	
4 + 5	4	
5 + 4	3	
T stage		
1a	1	
1c	19	
2a	12	
2b	4	
2c	10	
3a	3	
3b	5	
Risk group		
Low risk	4	
Intermediate risk	31	
High risk	20	
Positive cores (%)	34.8	25.8
Hormonal therapy		
Yes	37	
No	18	

Values represent mean or number

iPSA, initial prostate-specific antigen; SD, standard deviation

**Fig. 1:**
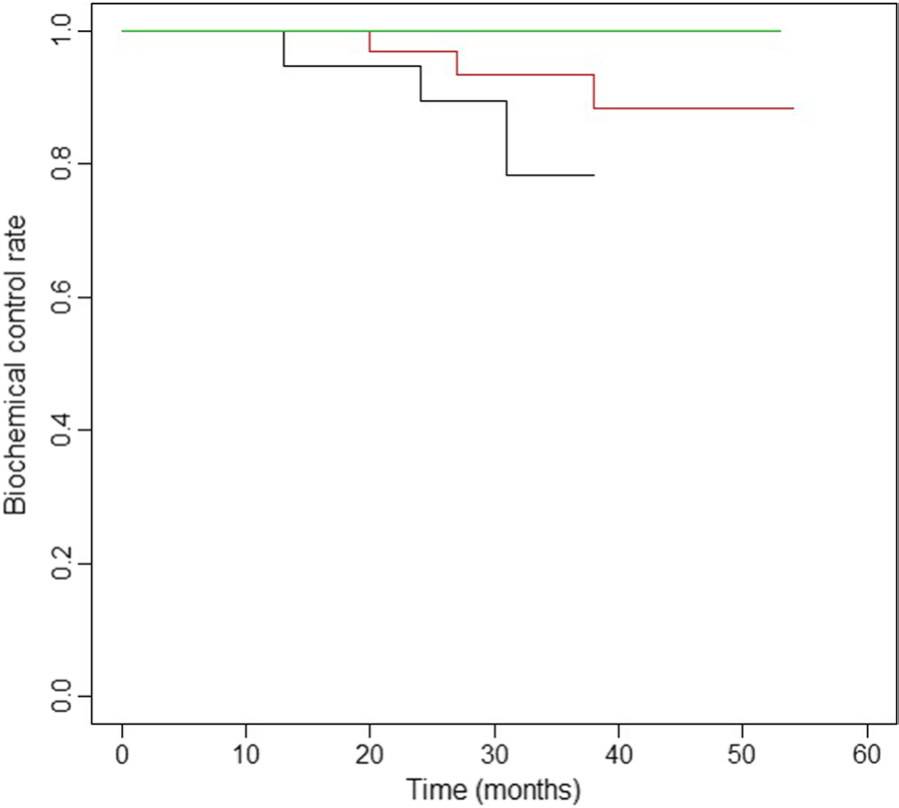
Biochemical recurrence-free survival rates by risk group. Biochemical recurrence-free survival rates for low- (green), intermediate- (red), and high-risk patients (black)

Six patients showed biochemical recurrence during follow-up. Among those six patients, two patients developed bone metastasis, one patient had lymph node and lung metastases, and one patient received salvage hormonal therapy. One patient showed bone metastasis before PSA recurrence. One patent died of gastric cancer (Table 3). The 3-year biochemical control rate was 89.8% (95% confidence interval [CI] 81.6–98.8%) for the overall cohort, including 100% (95%CI, NA) for low-risk patients, 93.3% (95%CI, 84.8–100%) for intermediate-risk, and 78.3% (95%CI, 57.8–100%) for high-risk patients. The corresponding rate of clinical no-evidence-of-disease survival was 92.4% (95%CI, 85.6–99.9%) for the overall cohort, including 100% (95%CI, NA) for low-risk patients, 93.4% (95%CI, 85.0–100%) for intermediate-risk patients, and 89.2% (95%CI, 76.0–100%) for high-risk patients (Fig. 1).

**Table 3:** Characteristics of patients showing recurrence

Case	T stage	iPSA	Gleason score	Risk category	Hormonal therapy (months)	PSA failure (months)	Clinical failure (months)	Site of recurrence	Status at last follow-up
1	2a	35.29	4 + 5	H	45	31	30	Bone	Alive
2	3b	82.98	5 + 4	H	32	13	14	Bone	Alive
3	1c	14.78	3 + 4	I	NA	38	42	Hormone treatment	Alive
4	1c	11.4	3 + 4	I	NA	20	24	Lymph node, lung	Alive
5	1c	10.9	4 + 4	H	NA	24	NA	NA	Alive
6	1c	13.291	3 + 4	I	NA	27	NA	NA	Alive
7	2c	7.5	3 + 3	I	10	NA	12	Bone	Alive

iPSA, initial prostate-specific antigen

Table 4 shows crude rates of acute and late toxicities. Acute grade 2 genitourinary (GU) and gastrointestinal (GI) toxicities were observed in 5 (9%) and 6 (11%) patients, respectively. No grade 3 or higher acute toxicities were observed. Late grade 2 GU and GI toxicities were observed in 7 (13%) and 4 (7%) patients, respectively. Grade 3 GU and GI toxicities were seen in 1.8% of patients each.

**Table 4:** Acute and late toxicities

	Acute						Late					
	G1		G2		G3		G1		G2		G3	
RTOG												
GU	23	(41.8%)	5	(9.1%)	0	(0.0%)	27	(49.1%)	7	(12.7%)	1	(1.8%)
GI	13	(23.6%)	6	(10.9%)	0	(0.0%)	17	(30.9%)	4	(7.3%)	1	(1.8%)
Miction pain	5	(9.1%)	1	(1.8%)	0	(0.0%)	12	(21.8%)	0	(0.0%)	0	(0.0%)
Frequency	19	(34.5%)	3	(5.5%)	0	(0.0%)	16	(29.1%)	6	(10.9%)	1	(1.8%)
Urinary incontinence	0	(0.0%)	0	(0.0%)	0	(0.0%)	0	(0.0%)	0	(0.0%)	0	(0.0%)
Retention	8	(14.5%)	2	(3.6%)	0	(0.0%)	6	(10.9%)	2	(3.6%)	0	(0.0%)
Hematuria	0	(0.0%)	0	(0.0%)	0	(0.0%)	10	(18.2%)	0	(0.0%)	1	(1.8%)
Stricture	0	(0.0%)	0	(0.0%)	0	(0.0%)	0	(0.0%)	0	(0.0%)	0	(0.0%)
Proctitis	6	(10.9%)	2	(3.6%)	0	(0.0%)	3	(5.5%)	0	(0.0%)	0	(0.0%)
Fecal incontinence	2	(3.6%)	1	(1.8%)	0	(0.0%)	1	(1.8%)	0	(0.0%)	0	(0.0%)
Diarrhea	7	(12.7%)	1	(1.8%)	0	(0.0%)	3	(5.5%)	0	(0.0%)	0	(0.0%)
Rectal hemorrhage	5	(9.1%)	2	(3.6%)	0	(0.0%)	15	(27.3%)	5	(9.1%)	1	(1.8%)

GU, genitourinary toxicity; GI, gastrointestinal toxicity

RTOG, Radiation Therapy Oncology Group

**Fig. 2:**
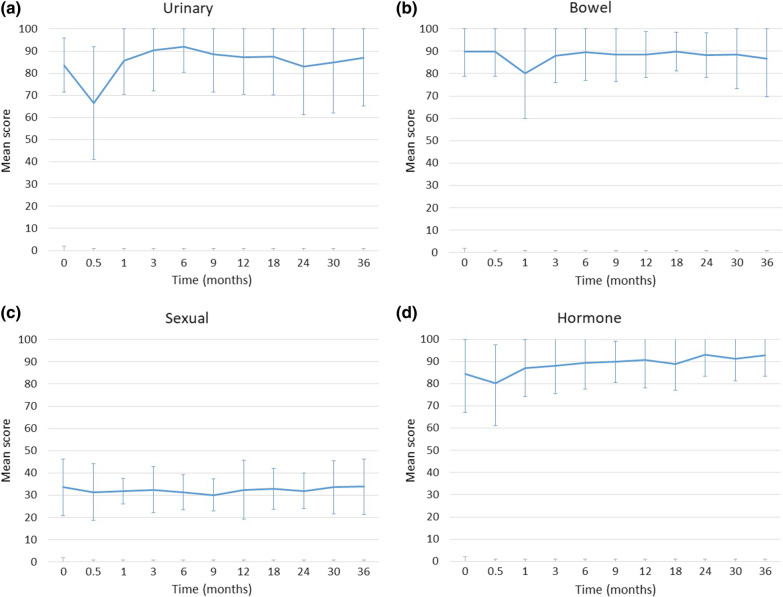
Patient-reported outcomes as assessed by Expanded Prostate Cancer Index Composite. A temporary drop and subsequent recovery are seen within the first 3 months after treatment for urinary (**a**) and bowel (**b**) functions. No significant changes were observed for sexual (**c**) and hormone (**d**) function. Error bars represent standard deviations

Figure 2 shows patient-reported outcomes as assessed by EPIC. Mean EPIC urinary function scores declined from 83.7 at baseline to 66.6 in 2 weeks (mean change from baseline, − 17.1), and returned to near baseline at 1 month (Fig. 2a). Mean EPIC bowel function scores declined from 89.9 at baseline to 80.2 in one month (mean change from baseline, − 9.7), and returned to near baseline in the third month (Fig. 2b). Regarding sexual and hormonal functions, no significant changes were observed (Fig. 2c, d).

## Discussion

The efficacy and safety of prostate SBRT using > 5 Gy per fraction have already been demonstrated by randomized control trials [13, 14], long-term pooled study [15], and meta-analysis [16]. In addition, the current version of the National Comprehensive Cancer Network (NCCN) guideline lists SBRT as a standard option for localized prostate cancer with all risk categories.

Although in current clinical practice the majority of SBRT for prostate cancer uses 5 fractions because all dose-escalation trials used 5 fractions [17–21], no optimal schedule has been determined for prostate SBRT. In this regard, our 4-fraction schedule could offer several benefits over a 5-fraction schedule, as described in previous reports [7]. In brief: 1) additional tumor control effects might be obtained for the same level of toxicity; 2) basically, a 4-fraction schedule showed no treatment carry-over from the previous week even when national holidays were inserted in addition to Saturday and Sunday; 3) although a difference of one fraction might only be relatively small for a single patient, the difference in total cost would not be negligible for high-volume centers such as academic institutes.

Regarding tumor control with our 4-fraction schedule, we believe that our results were comparable to those from other studies. Because most of our patients with recurrence showed distant metastases at relatively early time points after treatment (Table 3), those patients may have had distant metastasis in the staging phase and the local control rate in our study was comparable to the level achieved in other SBRT studies and conventional fractionation studies.

Regarding toxicity, we believe that our results were acceptable considering pioneering studies that reported relatively high incidences of Grade 2 and Grade 3 toxicities [22–29]. However, compared to current standard dose regimens such as 36 Gy in 5 fractions, our toxicity with 3 years of follow-up might have been slightly more severe, particularly for GI toxicities. For example, Katz reported frequencies of 4% and 0% for late GI Grade 2 and 3 toxicities, respectively, from 10-year results of 230 patients treated with 35–36.35 Gy in 5 fractions [2]. In addition, the American Society for Radiation Oncology, American Society of Clinical Oncology, and American Urological Association guidelines recommend doses between 35 Gy and 36.25 Gy in 5 fractions, and doses above 36.25 Gy are not suggested outside the setting of clinical trials due to the risk of late toxicities [30]. The regimen of 36 Gy in 4 fractions (equivalent dose in 2-Gy fractions: EQD = 86.4 Gy), is equivalent to 39.6 Gy in 5 fractions with an assumption of α/β = 3, and it seems that the regimen of the current study might exceed the recommended range. Therefore, our current protocol was changed to 32 Gy in 4 fractions for low- and intermediate-risk patients and 34 Gy in 4 fractions for high-risk patients.

To the best of our knowledge, only a few studies have reported a 4-fraction schedule for prostate SBRT. Fuller et al. reported their experience with 38 Gy in 4 fractions using brachytherapy-like dosimetry [4, 5], achieving promising tumor control rates. However, relatively severe toxicity rates such as 3–6% for grade 3 GU toxicities were reported. Kang et al. described their experience with 32–36 Gy in 4 fractions and median follow-up of 4 years. Biochemical control rates were sufficient, at 100%, 100%, and 90.8% for low-, intermediate-, and high-risk patients as of final follow-up. However, they also reported relatively severe toxicities after 36 Gy in 4 fractions and changed the protocol to 34 Gy in 4 fractions [6].

Several limitations to this study should be kept in mind, including the relatively small number of patients and immature follow-up, especially with regard to oncological outcome and late toxicities. Longer follow-up might be needed for proper assessment of the clinical outcomes and late toxicity profile, because twenty high-risk patients were participated in the present study. They initially underwent 6 months of neoadjuvant ADT, and adjuvant ADT was continued adjuvant ADT for 36 months after completion of radiotherapy, which is equal to the median follow-up period.

In the future, cases of prostate cancer in Japan will increase further. In particular, geriatric assessment increases the indications for radiotherapy rather than surgery. Workload issues are expected to arise if all such cases are treated with conventional fractionated radiotherapy. SBRT would address this problem. SBRT using 4 fractions represents a step forward, and further developments and improvements are expected.

## Conclusions

Our phase II trial of 36 Gy in 4 fractions confirmed the efficacy and safety of this regimen for patients with localized prostate cancer, although this regimen showed slightly more severe toxicities than current dose standards, such as 36 Gy in 5 fractions.

## Data Availability

Data sharing is not applicable to this article, as no datasets were generated or analyzed during the current study.

## Abbreviations

SBRT: Stereotactic body radiotherapy
CT: Computed tomography
CTV: Clinical target volume
PTV: Planning target volume
EPIC: Expanded Prostate Cancer Index Composite
GI: Gastrointestinal
GU: Genitourinary
QOL: Quality of life
